# Mechanistic insights into the clinical Y96D mutation with acquired resistance to AMG510 in the KRAS^G12C^

**DOI:** 10.3389/fonc.2022.915512

**Published:** 2022-08-10

**Authors:** Haiming Zhuang, Jigang Fan, Mingyu Li, Hao Zhang, Xiuyan Yang, Ligen Lin, Shaoyong Lu, Qing Wang, Yaqin Liu

**Affiliations:** ^1^ Department of Pathophysiology, Key Laboratory of Cell Differentiation and Apoptosis of Chinese Ministry of Education, Shanghai Jiao Tong University, School of Medicine, Shanghai, China; ^2^ Zhiyuan Innovative Research Center, Shanghai Jiao Tong University, Shanghai, China; ^3^ State Key Laboratory of Quality Research in Chinese Medicine, Institute of Chinese Medical Sciences, University of Macau, Macao, Macao SAR, China; ^4^ Medicinal Chemistry and Bioinformatics Center, Shanghai Jiao Tong University, School of Medicine, Shanghai, China; ^5^ Oncology Department, Xin Hua Hospital Affiliated to Shanghai Jiao Tong University, School of Medicine, Shanghai, China

**Keywords:** KRAS, G12C, molecular dynamics simulations, AMG510, drug resistance

## Abstract

Special oncogenic mutations in the RAS proteins lead to the aberrant activation of RAS and its downstream signaling pathways. AMG510, the first approval drug for KRAS, covalently binds to the mutated cysteine 12 of KRAS^G12C^ protein and has shown promising antitumor activity in clinical trials. Recent studies have reported that the clinically acquired Y96D mutation could severely affect the effectiveness of AMG510. However, the underlying mechanism of the drug-resistance remains unclear. To address this, we performed multiple microsecond molecular dynamics simulations on the KRAS^G12C^−AMG510 and KRAS^G12C/Y96D^−AMG510 complexes at the atomic level. The direct interaction between the residue 96 and AMG510 was impaired owing to the Y96D mutation. Moreover, the mutation yielded higher flexibility and more coupled motion of the switch II and α3-helix, which led to the departing motion of the switch II and α3-helix. The resulting departing motion impaired the interaction between the switch II and α3-helix and subsequently induced the opening and loosening of the AMG510 binding pocket, which further disrupted the interaction between the key residues in the pocket and AMG510 and induced an increased solvent exposure of AMG510. These findings reveal the resistance mechanism of AMG510 to KRAS^G12C/Y96D^, which will help to offer guidance for the development of KRAS targeted drugs to overcome acquired resistance.

## Introduction

The RAS proteins, as a group of small GTPases, play a critical role in transduction of intracellular signaling pathways controlling cell proliferation, differentiation and apoptosis (1). It cycles between the GTP-bound active state and GDP-bound inactive state, acting as a binary switch. The cycle regulates downstream signaling pathways such as Raf/MEK/ERK pathway and PI3K/AKT/mTOR pathway (2). The activation of RAS relies on guanine nucleotide exchange factors (GEFs) that catalyze the exchange of GDP by GTP. In contrast, RAS is inactivated by its intrinsic GTPase activity and can be assisted by GTPase-activating proteins (GAPs) (3). Specific mutations of RAS inhibit its inactivation and induces the aberrant activation of downstream pathways, leading to oncogenesis (4, 5). Accumulating evidence indicates that oncogenic RAS mutations participate in approximately 30% of human cancers and induce over 1 million deaths per year worldwide (6, 7). There are four closely related RAS isoforms: HRAS, KRAS-4A, KRAS-4B and NRAS. Among them, KRAS mutations account for 85% of observed RAS mutations in cancer (8). KRAS mutations are dominated by single-base missense mutations, and 98% of which are found at three mutation hotspots: G12, G13, and Q61 (6, 9). Compared with other isoforms, KRAS is the only RAS isoform where the position 12 mutations are predominant (10). GTPase cycle is based on the conformational transition of RAS. Three functional elements in the effector lobe (residues 1–87) of Ras, including P-loop (residues 10–17), switch I (residues 32– 38), and switch II (residues 59–76), are pivotal for such conformational changes (11). With GTP bound, the three elements form the closed conformation of the catalytic site residing in the active state, while they adopt the open conformation after GTP hydrolysis residing in the inactive state (12). In the active state, Tyr32 on the switch I domain is in the “up” conformation and the α2-helix on the switch II domain is positioned in the “inward” conformation towards the α3-helix, denoted as “α2-helix_in_” (13). In contrast, the inactive state adopts the “Tyr32_down_” and “α2-helix_in_” conformation. To be noted, specific mutations in the switches can also affect the conformational transition of RAS (14). For example, D33E in the switch I can stabilize the open conformation of RAS even with GTP binding (15).

Previously, Ras was considered as ‘‘undruggable” due to its picomolar affinities towards GDP/GTP in the orthosteric site and the lack of suitable binding sites along the surface (16–18). However, many of the newly proposed drug design strategies including reversed allosteric communication are gradually turning the ‘‘undruggable” RAS “drugged” (19–27). The most exciting advance is the advent of KRAS^G12C^ inhibitors (28–31). They bind covalently to the mutated cysteine 12 and lock the KRAS in the inactive state, thereby inhibiting the hyperactivation of downstream signaling (32–34). AMG510 (Sotorasib), one of these covalent inhibitors, binds irreversibly with the allosteric binding pocket of KRAS^G12C^, which mainly consists of switch II and α3-helix, termed as the switch II pocket (35). AMG510 has shown particularly promising anticancer activity in clinical trials for KRAS^G12C^ mutant patients and is the first approval drug for KRAS targeted therapy by FDA (36–39). Drug resistance has always been a great challenge to the clinical use of drugs (40, 41) and the clinical Y96D acquired resistance mutation to AMG510 has been observed (42, 43). Cell viability assays showed that relative to the KRAS^G12C^ expressing controls, cells expressing the double KRAS^G12C/Y96D^ mutation showed marked resistance to AMG510, with an IC_50_ value shifting >100-fold. The crystal structure of AMG510−KRAS^G12C^ complex implies that the Y96D mutation disrupts the water-mediated hydrogen bond between Y96 and the carboxyl group of AMG510.

However, the water-mediated hydrogen bond between Y96 and the carboxyl group of AMG510 in the crystal structure seems relatively weak, hard to explain the significant divergence of IC_50_ induced by the mutation. Furthermore, crystal structures are a spatiotemporal average of the protein structure with crystal-packing contacts, which may not provide enough information on the conformational transition of proteins. Molecular dynamics (MD) simulations that explore protein conformational dynamics at the atomic level can be helpful to investigate the resistant mechanism of Y96D mutation. MD simulations have been applied to investigate the conformational dynamics of RAS proteins (44, 45). In recent work, we have used MD simulations to directly uncover biomolecular mechanisms and protein–ligand/protein recognitions (46–54). In addition, although previous studies have demonstrated that Y96 plays a critical role in AMG510 binding (55), no studies have shown how Y96D mutation affects the conformation of the KRAS protein. 

Here, we performed an extensive large-scale MD simulations of the KRAS^G12C^−AMG510 and KRAS^G12C/Y96D^−AMG510 complexes to investigate the underlying mechanism of the additional Y96D mutation-based resistance to AMG510. The simulation revealed that the Y96D mutation impaired the van der Waals interaction between residue 96 and AMG510 and yielded higher flexibility and more coupled motion of the switch II and α3-helix, which led to the departing movement of the switch II and α3-helix. The departing motion induced the opening and loosening of the binding pocket, disrupting the interactions of KRAS−AMG510. Moreover, the opening of the binding pocket caused an increased solvent exposure of AMG510, which may yield the instability of AMG510. Collectively, this study revealed the underlying mechanism of the Y96D resistance mutation, which will help to offer guidance for the design of KRAS targeted drugs.

## Materials and methods

### System preparation

Two systems were performed, including KRAS^G12C^−AMG510 (denoted as “G12C”) and KRAS^G12C/Y96D^−AMG510 (denoted as “G12C/Y96D”). The crystal structure of KRAS^G12C^−AMG510 complex (PDB ID: 6OIM) (43) was selected as the initial structure. The missing residues of the KRAS protein were added using the MODELLER program. The Y96D mutation was conducted using the Discovery Studio 2019.

### MD simulations

The force field parameter of AMG510 was generated using the antechamber program of Amber. The systems were prepared with the LEaP program using ff14SB force field to describe the AMG510−protein complexes (56). The prepared complex was solvated to a truncated octahedron transferable intermolecular potential three point (TIP3P) water box (57) and Na+ and Cl- were added to neutralize and simulate normal saline environment. Two rounds of minimization were performed. The first round contained 5000 steps maximum minimization cycles with the complex fixed and the second round contained 10,000 steps maximum minimization cycles with no constraints. Subsequently, systems were heated from 0 to 300 K within 300 ps, followed by 700 ps equilibration running in a canonical ensemble (NVT). After all these preparations, 3 replicas of independent 1 μs simulations were performed with random velocities under isothermal isobaric (NPT) conditions for each system. For the long-range electrostatic interactions, the particle mesh Ewald method (58) was used. Covalent bonds involving hydrogens were restrained using the SHAKE method (59).

### Principal component analysis (PCA)

Principal component analysis (PCA), widely used in describing the kinetic process during simulation, is a technique that can transform a series of potentially coordinated observations into orthogonal vectors. Among these vectors, the first principal component (PC1) provides the dominant motions throughout simulation (60). PCs were generated based on coordinate covariance matrix of Cα atoms in the KRAS protein using every frame in all two systems and these collected frames were all projected on the PC1 and PC2.

### Dynamic cross-correlation matrix (DCCM) analysis

The DCCM of all protein Cα atoms was calculated to reflect the inter-residue correlations. The cross-correlation coefficient *C_i,j_* was calculated by:


[1] $$C_{i,j}=\frac{c\left(i,j\right)}{\sqrt{c\left(i,i\right)}\;\sqrt{c\left(j,j\right)}}$$

where *i* and *j* represent the *i* ^th^ and *j* ^th^ Cα atoms, respectively.

### Generalized correlation analysis

Generalized correlation (*GC_i,j_*) analysis was applied to calculate the correlated motion. Comparing with the traditional DCCM analysis, *GC_i,j_* analysis has the advantage of capturing the non-liner correlations by calculating the correlations independently on the relative orientation of the atomic fluctuations. To reflect how much information of one atom’s position is provided by another, Mutual Information (MI) was introduced and calculated by:


[2] $$MI\left[x_{i},x_{j}\right]=\int \int p\left(x_{i},x_{j}\right)ln\frac{p\left(x_{i},x_{j}\right)}{p\left(x_{i}\right)p\left(x_{j}\right)}dx_{i}dx_{j}$$

The right side of equation can be related to the more widely known measure of entropy which calculated by:


[3] H[x]=∫p(x)lnp(x)dx

To calculate based on the correlation between pairs of atoms:


[4] $$MI\left[x_{i},x_{j}\right]=H\left[x_{i}\right]+H\left[x_{j}\right]-H\left[x_{i},x_{j}\right]$$

[*x_i_*], was further related to a more intuitive Pearson-like correlation coefficient *GC_i,j_* which can be calculated by:


[5] $$GC_{ij}\;={\left\{1-e^{-\frac{2MI\left[x_{i},x_{j}\right]}{d}}\right\}}^{-\frac{1}{2}}$$

where *d* represents the dimensionality of *x_i_* and *x_j_*, which is 3 in our study. *GC_i,j_* calculation was done by g_correlation tool in Gromacs 3.3 (61) with the coordinates of Cα atoms in each residue as input.

### Dynamic network analysis

Dynamic network analysis was performed to reflect the motion connection using the Network View plugin in VMD (62). In our analysis, the Cα atoms of KRAS were selected as nodes to represent their corresponding residues. Edges were drawn between nodes whose distances are within a cutoff of 4.5 Å for at least 75% of simulation time. The edge between nodes was calculated using:


[6] $$d_{i,j}=-\text{log}\left(\left|C_{i,j}\right|\right)$$

where *i* and *j* represent the two nodes. Additionally, community, which means a combination of residues whose connections are stronger was calculated using the Girvan-Newman algorithm (63) through the communities program of VMD.

### Markov state model (MSM)

Markov state model (MSM) is a mathematical framework to describe the dynamics of time-series data (64). In this analysis, MSM was used to differentiate conformational states throughout simulation and help to extract representative structures from each cluster. All MSM calculations were performed using the PyEMMA software. MSM transition matrix was first calculated based on the probability of transition between different states. Implied timescale (ITS) test was performed to check the Markovian property and choose the proper lag time(τ). ITS as a function of τ can be calculated by:


[7] $$t_{i}=-\frac{\tau }{\text{ln}\left|\lambda_{i}\left(\tau \right)\right|}$$

where *λ_i_* means the eigenvalue gotten from MSM transition matrix of the i^th^ process. In implied timescale plot, each curve represents an average transition time in one process. When the curve becomes approximately constant, the corresponding lag time is appropriate for following analysis and the system is with Markovian property. In this study, the lag time was set as 1 ns.

Every frame of each system was projected on the free energy plot according to the characteristic vectors (CV). Then, K-means-algorithm was applied to cluster the two-dimensional conformations into 100 microstates in each system. Based on these 100 microstates, PCCA+ algorithm was performed to divide the microstates into different clusters. With the divided clusters, the Chapman–Kolmogorov test was computed to testify the property of MSM. The frames near the K-centers (microstates) belonging to the certain state in energy landscape were extracted to constitute a new trajectory to represent the state. The structure that has the smallest root-mean-square deviation (RMSD) with frames in the representative trajectories was chosen as the representative structure. For only the states in the energy basin of free-energy landscape were analyzed, the first cluster in the G12C system was not analyzed and the two clusters differentiated by MSM in the G12C/Y96D system were combined.

## Results

### Y96D mutation affects conformational dynamics of KRAS

To investigate the effect of mutation on overall conformational dynamics of KRAS protein, we calculated the Cα atoms root-mean-square deviation (RMSD) of the KRAS protein relative to the initial structure. The results indicated that both systems reached equilibrium after 100 ns of simulation (**Supplementary Figure 1A**). The RMSD values were 1.40 ± 0.16Å for the G12C system and 1.39 ± 0.10Å for the G12C/Y96D system. The similarity of the RMSD values between the two systems indicated the minor effect of the Y96D mutation on overall protein conformational dynamics.

**Figure 1:**
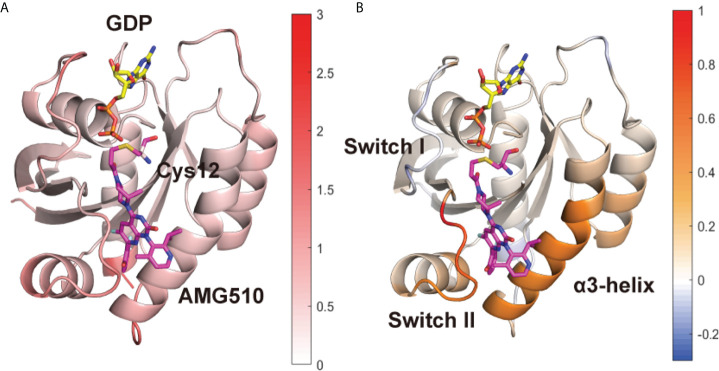
Conformational dynamics of KRAS protein. **(A)** The averaged RMSF values of each residue are plotted on the 3D structures of the KRAS protein in the G12C system. **(B)** The substation of RMSF values of the G12C/Y96D system from its counterpart in the G12C system. Positive regions (red) stand for higher RMSF values, whereas negative regions (blue) represent lower RMSF values.

To further uncover the influence of mutation on local conformational dynamics, the root-mean square fluctuation (RMSF) of each residue (represented by the Cα atom) was calculated (**Supplementary Figure 1B**). To better show the differences, we subtracted the RMSF value of the G12C/Y96D system from its counterpart in the G12C system (**Figure 1A**) and projected the values on the protein structure. Notably, the RMSF of the switch II and α3-helix in the G12C/Y96D system was higher than that in the G12C system (**Figure 1B**), suggesting that the Y96D mutation induced higher plasticity of the switch II and α3-helix. Because AMG510 binds to the switch II pocket that is mainly composed of the switch II and α3-helix, the increased plasticity of the switch II and α3-helix can be ascribed to their impaired interaction with AMG510 induced by the mutation. Additionally, the RMSF value of the switch I in the G12C/Y96D system was slightly lower than that in the G12C system, indicating that the Y96D mutation may stabilize the switch I region of KRAS.

### Y96D mutation induces conformational transition of KRAS

**Figure 2:**
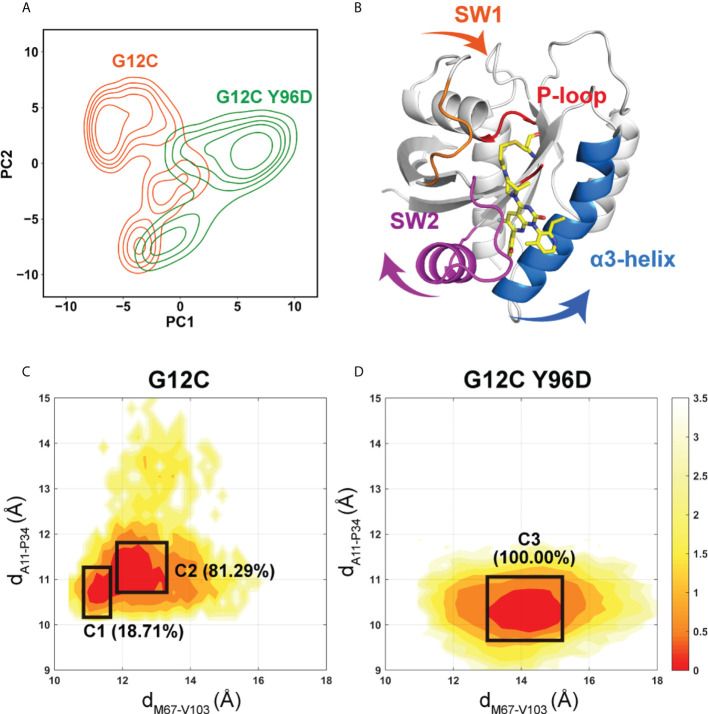
Global conformational transition of the KRAS protein. **(A)** Projections of the first and second principal components (PC1 vs PC2) from MD simulations of the G12C system (orange) and the G12C/Y96D system (green). **(B)** The motion along the PC1. Conformational landscapes generated using the d_M67-V103_ (distance from the M67 Cα atom to the V103 Cα atom) and d_A11-P34_ (distance from the A11 Cα atom to the P34 Cα atom) order parameters in the G12C system **(C)** and the G12C/Y96D system **(D)**.

To characterize the global conformational transition between the two systems, principal component analysis (PCA) was performed. We projected all MD snapshots from simulation of each system on the two-dimensional plot according to the first two principal components (PC1 and PC2) (**Figure 2A**) and the conformational motion along the PC1 was shown on the KRAS protein (**Figure 2B**). A significant increase of PC1 value was observed in the G12C/Y96D system compared with the G12C system. Along the PC1, the switch I moved towards the P-loop, and the switch II and α3-helix underwent the departing motion. Because the switch II and α3-helix constitute the most of the AMG510 binding pocket, this departing motion represented the opening of the binding pocket, reflecting the transition from the “closed” to “opening” motions.

To further probe the conformational transition induced by the Y96D mutation, we projected all MD trajectories onto the two-dimensional surface according to the distances from the M67 Cα atom to the V103 Cα atom and from the A11 Cα atom to the P34 Cα atom (**Figures 2C, D**). D_M67-V103_ represents the distance between the switch II and α3-helix while d_A11-P34_ represents the distance between the switch I and P-loop (**Supplementary Figure 2**). In agreement with the motion along the PC1, the G12C/Y96D system had larger d_M67-V103_ and smaller d_A11-P34_ distances, indicating that the Y96D mutation induced the departure of the switch II and α3-helix and the approaching of the switch I to the P-loop. The range of d_M67-V103_ parameter was much broader in the G12C/Y96D system than that in the G12 system, which was in accordance with the increased plasticity of the switch II and α3-helix induced by the Y96D mutation shown in RMSF analysis. Similarly, the limited range of the d_A11-P34_ parameter in the G12C/Y96D system compared to the G12 system was consistent with the stabilization of the switch I induced by the Y96D mutation shown in RMSF analysis.

**Figure 3:**
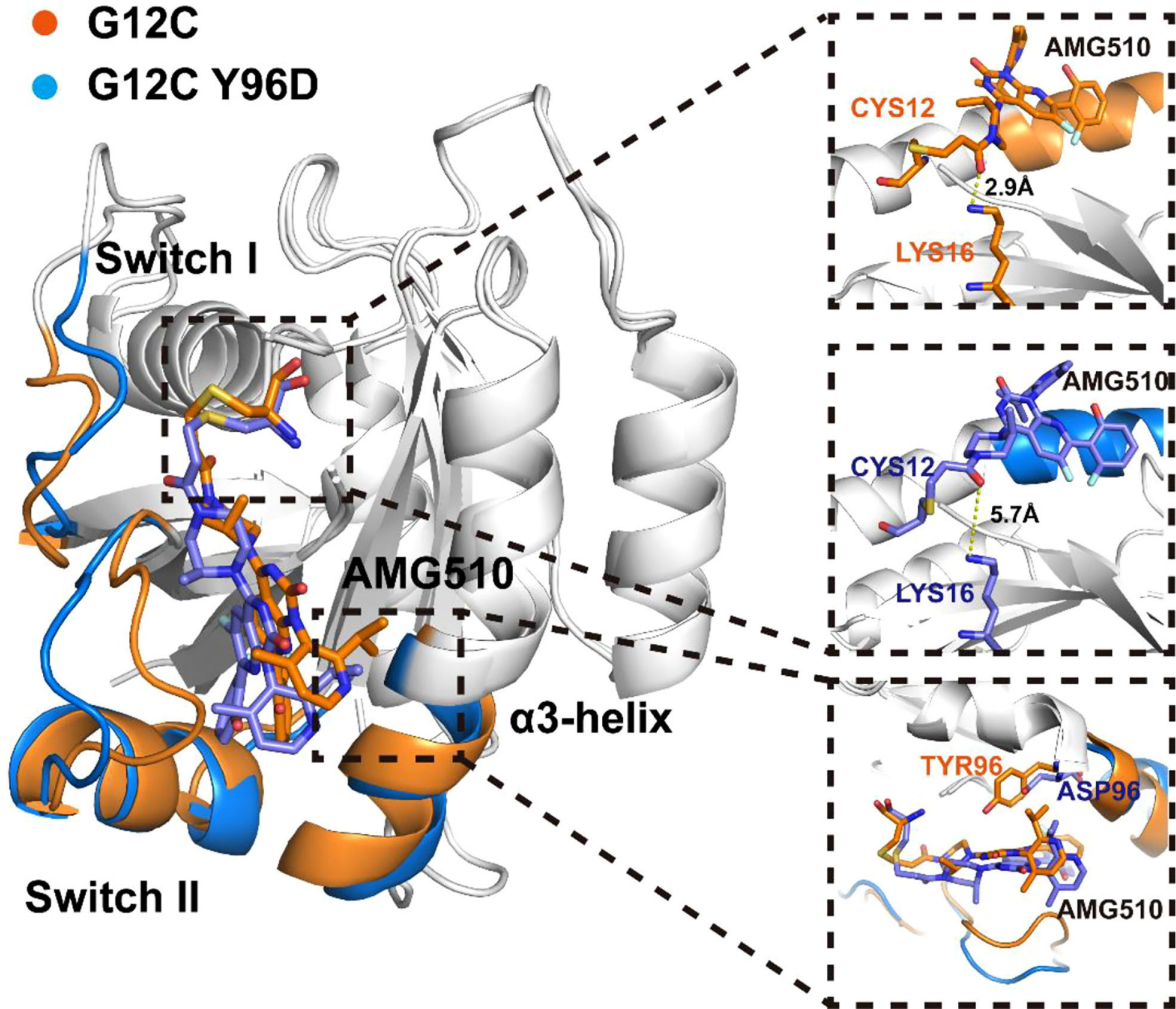
Superposition of the representative structures of the G12C system (orange) and the G12C/Y96D system (blue).

**Figure 4:**
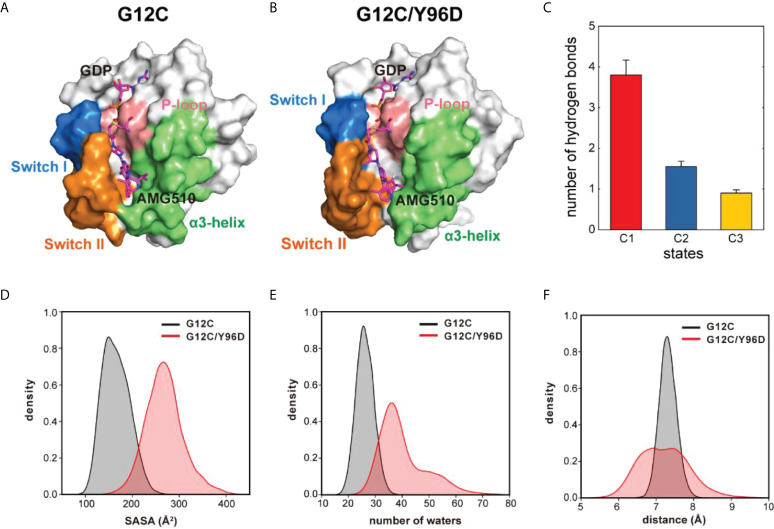
The Y96D mutation induced the opening and loosening of the binding pocket. **(A)** The surface representation of the representative structure of the G12C system. **(B)** The surface representation of the representative structure of the G12C/Y96D system. **(C)** The number of hydrogen bonds formed between the switch II and α3-helix in each state. **(D)** SASA of AMG510 in the G12C system (black) and the G12C/Y96D system (red). **(E)** Number of waters in the 5.4 Å watershell of AMG510 in the G12C system (black) and the G12C/Y96D system (red). **(F)** The distance between the CB atom of residue 96 and the centroid of azaquinozoline of AMG510 in the G12C system (black) and the G12C/Y96D system (red).

Markov state model (MSM) was used to differentiate the conformational states in the free-energy landscape, and only the states in the energy basin of free-energy landscape were analyzed. To testify the Markovian property, the implied timescale test and the Chapman−Kolmogorov test were applied (**Supplementary Figure 3**). The proportion, the representative trajectory, and the structure of each state were calculated based on the MSM. In the G12C system, with the increase of the d_M67-V103_ parameter, two states were observed: C1 and C2 (**Figure 2C**). The C2 state was the dominant state. In the G12C/Y96D system, only one state (C3) was observed (**Figure 2D**). In the G12C/Y96D system, the d_M67-V103_ parameter of the centroid of the C3 state in the free-energy landscape was larger than that of the C2 and C3 states in the G12C system. This indicated that the Y96D mutation could induce the departure of the switch II and α3-helix. To further illustrate the conformational transition, we superimposed the representative structures of the dominant state in the G12C system (C2) and the dominant state in the G12C/Y96D system (C3) (**Figure 3**). The departure of the switch II and α3-helix was observed and the motion was particularly obvious in the loop region of the switch II, the key region that interacts with AMG510. The surface representations of the representative structures showed that the direct interaction between the switch II and α3-helix was disrupted, which led to the opening of the binding pocket (**Figures 4A, B**).

To further investigate the transition between the switch II and α3-helix, we calculated the hydrogen bonds formed between these two regions in the C1, C2 and C3 states (**Figure 4C**). The number of hydrogen bonds between the switch II and α3-helix decreased from the C1 to the C3, indicating that the departing motion of the switch II and α3-helix would impair the switch II and α3-helix interaction from the C1 to C3. The decreased hydrogen bonds occupancy between the switch II and α3-helix in the G12C/Y96D mutation further proved that the G12C/Y96D mutation could induce the departing motion of the switch II and α3-helix, which impaired the interaction between these two regions and led to the opening of the binding pocket (**Supplementary Table 1**). Taken together, the departure of the switch II and α3-helix induced by the Y96D mutation disrupted the interaction between these two regions and led to the loosening and opening of the binding pocket.

### Y96D mutation induces the increased solvent exposure of AMG510

The solvent accessible surface area (SASA) of AMG510 was calculated to quantify the solvent exposure of AMG510. The SASA value in the G12C/Y96D system was significantly higher than that in the G12C system (**Figure 4D**), indicating a more loosened wrapping of binding pocket. Because SASA was limited to reflect the real solvent exposure, we calculated the number of waters in the solvent shell that wrapped AMG510 and the shell radius was set as 5.0 Å. With the Y96D mutation, the number of waters in the solvent shell increased (**Figure 4E**), suggesting an increased solvent exposure of AMG510 induced by the Y96D mutation. The extended range of the number of waters in the solvent shell in the G12C/Y96D system implied the instability of AMG510 binding pocket. The increased solvent exposure can be ascribed to the observation that the Y96D mutation promoted the departure of the switch II and α3-helix and induced the loosening and opening of binding pocket. The closed binding pocket could protect AMG510 from water solvent attack in the G12C system, while such protection was disturbed in the G12C/Y96D system, which led to the decreased binding affinity.

### Y96D mutation impairs the interaction between KRAS and AMG510

Although AMG510 covalently binds to Cys12 of KRAS, other residues also play a significant role in the stable binding (65). One stable hydrogen bond was observed between Lys16 and the carbonyl oxygen of AMG510 whose occupancy was 83.76% in the G12C system while it was 7.10% in the G12C/Y96D system, indicating that the Y96D mutation disrupted the hydrogen bond between Lys16 and AMG510. The superimposition of representative structures of the two systems validated the disruption of the hydrogen bond in the G12C/Y96D system (**Figure 3**). Crystal structure shows water-bridged hydrogen bonds between Tyr96 and AMG510. The water-bridged hydrogen bonds were calculated to investigate the effect of the Y96D mutation on water bridges (**Supplementary Table 2**). The water-bridged hydrogen bond between AMG510 and Gln99 was the most stable water-bridged interaction between AMG510 and protein whose occupancy was 28.43% in the G12C system and 23.60% in the G12C/Y96D system. Interestingly, two additional water-bridged hydrogen bonds were strengthened in the Y96D mutation. One was formed with the mutated Asp96 and the other was formed with Lys16 which formed stable hydrogen bonds with AMG510 in the G12C system. Moreover, no conserved water bridges were observed for the highest occupancy of waters, implying that these water-bridged hydrogen bonds were delicate and may not the key interaction in keeping AMG510 binding.

To further investigate the interaction between AMG510 and KRAS, we calculated the linear interaction energy between key residues in the binding pocket and AMG510. The residues whose linear interaction energy having obvious variation between two systems were shown in **Table 1** and the electrostatic energy contribution and van der Waals energy contribution were shown, respectively. The van der Waals energy contribution from the residue 96 significantly decreased in the G12C/Y96D mutant, indicating that the Y96D mutation disrupted the van der Waals interaction between residue 96 and AMG510. By superimposing the representative structures of the G12C and the G12C/Y96D systems, we observed that the phenylol group of Tyr96 pointed directly to AMG510 while the mutated Asp96 was distant from AMG510 to form direct interaction (**Figure 3**). The distance between the CB atom of residue 96 and the centroid of azaquinozoline of AMG510 was calculated to reflect the interaction between residue 96 and AMG510. The distance range was more limited in the G12C system than in the G12C/Y96D system, indicating a more stable interaction between residue 96 and AMG510 in the G12C system (**Figure 4F**). The linear interaction energy of other key residues also decreased to varied degrees in the G12C/Y96D system and these key residues are concentrated at the loop of the switch II and α3-helix, consistent with the conformational transition induced by the Y96D mutation that the departure of the switch II and α3-helix from AMG510.

**Table 1:** Linear interaction energy (kcal/mol) between key residues and AMG510 in the two systems.

Residue	G12C		G12C/Y96D	
	Electrostatic energy (kcal/mol)	Van der Waals energy (kcal/mol)	Electrostatic energy (kcal/mol)	Van der Waals energy (kcal/mol)
Tyr96/Asp96	-1.53 (0.01)	-8.38 (0.02)	-3.41 (0.06)	-2.84 (0.03)
Thr58	-2.86 (0.03)	-2.86 (0.01)	-0.40 (0.01)	-1.60 (0.02)
Gln61	-0.13 (0.03)	-3.89 (0.03)	-0.20 (0.03)	-3.13 (0.04)
Glu63	-1.92 (0.02)	-2.97 (0.03)	-0.92 (0.03)	-1.89 (0.03)
His95	0.37 (0.01)	-2.24 (0.01)	0.44 (0.01)	-1.57 (0.02)
Gln99	-2.06 (0.01)	-5.49 (0.02)	-0.55 (0.02)	-5.57 (0.03)

All numbers in parentheses represent standard deviations.

### Y96D mutation disturbs the correlated motions and community networks

**Figure 5:**
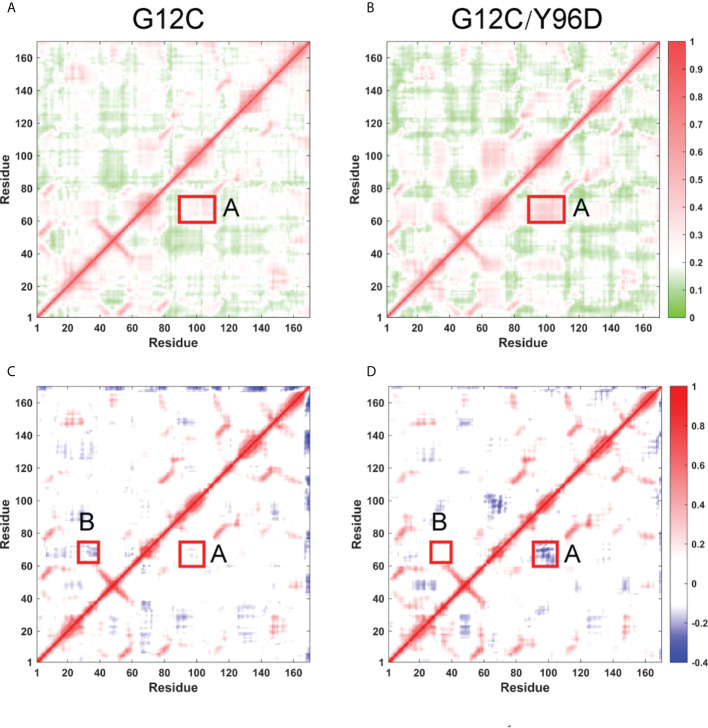
Inter-residue correlation of KRAS. Generallized residue correlation of the G12C system **(A)** and the G12C/Y96D system **(B)**. DCCM plot of the G12C system **(C)** and the G12C/Y96D system **(D)**. The significant correlation differences are highlighted with a rectangle in each panel. Regoin A represents the correlation between switch II and α3-helix while regoin B represents the correlation between switch I and switch II.

The above analysis indicated the coordinated motions of the switch I, switch II, and α3-helix. To further investigate the correlated motion, the generalized correlation analysis and dynamic cross-correlation matrix (DCCM) analysis were applied. The absolute value of coefficients is proportional to the correlation. The generalized correlation indicated an increased correlated motion between the switch II and α3-helix in the G12C/Y96D system (**Figures 5A, B**). In the dynamic cross-correlation matrix, the negative coefficients represent the motions towards the opposite direction and the positive coefficients represent the motions towards the same direction. Consistent with the generalized correlation, the opposite motion between the switch II and α3-helix (A region) was strengthened due to the Y96D mutation (**Figures 5C, D**), suggesting that the mutation yielded more coupled opposite motion of the switch II and α3-helix which led to their departing movement. Moreover, the Y96D mutation weakened the opposite motion between the switch I and switch II (B region) (**Figures 5C, D**), which was in line with the switch I motion observed in PCA analysis.

**Figure 6:**
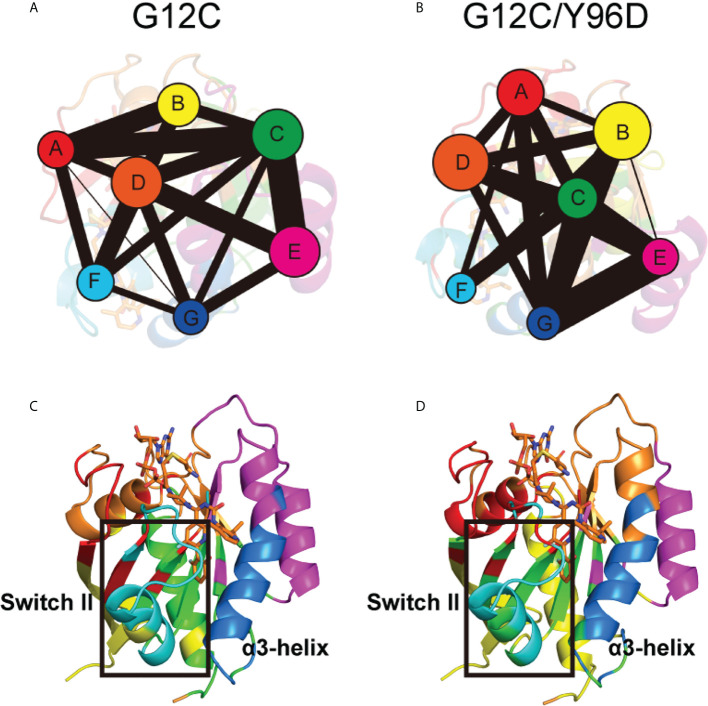
Community network of the KRAS protein. The community network in the G12C system **(A)** and the G12C/Y96D system **(B)**. Areas of the circles represent the numbers of residues in corresponding communities, and the widths of sticks connecting communities represent the intercommunity connections. Community composition in the G12C system **(C)** and the G12C/Y96D system **(D)**. The color of each community corresponds to **(A, B)**.

Community network analysis was performed to uncover the correlation network. Each community was showed as colored circles whose area is proportional to the number of residues it contains (**Figures 6A, B**). The intensity of inter-community connection was represented by the width of sticks connecting circles. In general, the Y96D mutation induced an obvious variation in community composition and connection. Switch II was mainly composed of Community F whose connection with Community D vanished in the G12C/Y96D system. For the cysteine 12 that covalently binds to AMG510 belonging to Community D, the vanished connection between Community D and Community F may suggest the damaged correlation between AMG510 and switch II. The optimal pathways that link Cys12 and Met67 (represents switch II) were calculated to prove the hypothesis. The shortest distance in the node map between Cys12 and Met67 in the G12C system is 244 Å, much shorter than that in the G12C/Y96D system (457 Å). Collectively, the community connection and the path length both reflected the impaired interaction between AMG510 and switch II. The connection between Community F (composed switch II) and Community G (composed α3-helix) also vanished in the G12C/Y96D system compared with the G12C system. This seemed to be in contradiction with the previous observation that the Y96D mutation yielded more coupled motion of the switch II and α3-helix. However, it should be noted that the Y96D mutation altered the composition of Community C that newly contained part of switch II (Q70, Y71 and T74) and α3-helix (V103) compared with the G12C system (**Figures 6C, D**). The coexistence of part of the switch II and α3-helix in the same community revealed a more correlated motion of the switch II and α3-helix in the G12C/Y96D system, consistent with previous observation. Overall, the Y96D mutation impaired the connection between the switch II and Cys12 that covalently binds to AMG510, and yielded more coupled motion of the switch II and α3-helix, which led to the departing movement of the switch II and α3-helix.

## Discussion

RAS proteins, as binary switches, control multiple intracellular signaling pathways. Specific oncogenic mutations disrupt the inactivation of RAS proteins and lead to the hyperactivation of downstream signaling pathways. Among them, mutations of KRAS account for 85% of observed RAS mutations in cancer and the mutation of residue 12 is one of the dominant mutations in KRAS (8). AMG510, the first drug for KRAS approved by FDA, covalently binds to Cys12 in the KRAS^G12C^ protein and has shown promising anti-cancer activity in clinical trials. The response that may induce drug resistance to these KRAS^G12C^ inhibitors can be divided into following categories, (i) mutations in KRAS, e.g., at G13, R68, H95, Y96, or multiple copies of KRAS^G12C^; (ii) mutations in genes other than KRAS; (iii) Transitioning to other cancers, e.g., transitioning from adenocarcinomas to squamous cell carcinomas (66). Among them, the resistance caused by the acquired Y96D mutation severely affects the effectiveness of AMG510. Here, using MD simulations, we investigated the resistance mechanism to AMG510, thereby providing guidance for optimization and design of targeted KRAS drugs.

The Y96D mutation disrupted the direct interaction between residue 96 and AMG510. By calculating the linear interaction energy, we found that the Y96D mutation disrupted the van der Waals interaction between residue 96 and AMG510. In contrast, the electrostatic interaction between residue 96 and AMG510 even slightly strengthened with the Y96D mutation. The paradoxical change can be ascribed to the substitution from the phenolic hydroxyl (Tyr) to the carboxyl group with negative charge (Asp). Thus, rebuilding the interaction between Asp96 and AMG510 by forming new electrostatic interaction might be an effective strategy to overcome the resistance. By superimposing the representative structures in the G12C system and the G12C/Y96D system, we found that the isopropyl group of AMG510 in the G12C system and the G12C/Y96D system were close to the carboxyl group of the mutated Asp96 (**Supplementary Figure 4**), just right for hydrogen bonding, implying that the substitution from the isopropyl group to a polar group such as amino might be viable to form additional electrostatic interaction with Asp96. The distance calculation between residue 96 and AMG510 also indicated a more stable interaction between them in the G12C system compared with the G12C/Y96D system. By forming the new electrostatic interaction mentioned above, the instability of AMG510 induced by the Y96D mutation could also be recovered.

The previously reported crystal structure of the KRAS^G12C^−AMG510 complex showed a bridged hydrogen bond mediated by two waters between Y96 and the carboxyl group of AMG 510. Tanaka et al. speculated that the Y96D mutation could impair the water-bridged hydrogen bond (43). However, the water-mediated hydrogen bond between residue 96 and AMG510 even strengthened in the simulation with the Y96D mutation. This can be ascribed to the disturbed interaction and the increased capacity to form hydrogen bond with the mutation from the phenolic hydroxyl (Tyr) to the carboxyl group with negative charge (Asp). Moreover, no conserved water bridges were observed for the waters participated in hydrogen bond bridge varied throughout simulation, indicating that these water-bridged hydrogen bonds were delicate and not the key interaction in keeping AMG510 binding. Such instability of water-bridged hydrogen bonds may be correlated with the mobility of solvation waters compared with crystal waters.

The Y96D mutation also affected the conformational dynamics and correlated motion of the KRAS protein. It is not surprising that the Y96D mutation changes the α3-helix’s dynamics because the Y96D mutation is located at the α3-helix. In addition, we found that the mutation affected the global dynamics of KRAS protein rather than only the α3-helix. The Y96D mutation yielded increased plasticity and more coupled motion of the switch II and α3-helix, which led to the departing movement of the switch II and α3-helix. The departing motion disrupted the interaction between the switch II and α3-helix and induced the opening and loosening of binding pocket. The “closed” to “opening” motion induced by the Y96D mutation further disrupted the interaction between the key residues in binding pocket and AMG510. Moreover, the opening of binding pocket caused an increased solvent exposure of AMG510, which may intensify the instability of AMG510. The communication between the switch II and α3-helix was also observed in other studies. These studies focused on the conformational changes in the switch II and α3-helix induced by mutations, which could impair the “activation” to “inactivation” conformational transition in GTPase circle of KRAS (67–69). Tanaka et al. found that KRAS^G12C/Y96D^ appeared to have higher basal activation than KRAS^G12C^ for a higher proportion of the active GTP-bound form of KRAS. From our research and other studies on the communication between the switch II and α3-helix, we can speculate that the increased activation of KRAS^G12C/Y96D^ was correlated with the coupled motion of the switch II and α3-helix. This is a possible direction in future research.

Research on the resistance to G12C covalent inhibitors identified many other acquired mutations in KRAS, which greatly affect the efficacy of KRAS^G12C^ targeted drugs, including Y96C, R68S and H95D/Q/R (70). Among them, H95D/Q/R mediated resistance to adagrasib (MRTX849) rather than sotorasib (AMG510) although His95 is part of the binding pocket of AMG510 (42). The linear interaction energy calculation in our study revealed that the contribution of His95 in AMG510 binding was very limited (**Table 1**), much less than key residues such as Tyr96.

Previously, Ras was considered as ‘‘undruggable” due to its picomolar affinities towards GDP/GTP in the orthosteric site and the lack of suitable binding pockets along the surface. Here, using fpocket software (71), we detected two new potential druggable pockets in the representative structures, which did not exist in the crystal structure. The two pockets can be detected in both the G12C system and the G12C/Y96D system with high druggability scores. The composition and location of these two pockets were shown in **Supplementary Figure 5**. This inspires future research aimed at the design of drugs targeting KRAS bound to these two new pockets.

Collectively, these results provided novel atomic-level insights to the resistance mechanism of the Y96D mutation to KRAS^G12C^ targeted drugs. First, they revealed the direct impairment of the Y96D mutation on the interaction between residue96 and AMG510. Second, they indicated that the Y96D mutation could promote the flexibility and the coupled motion of the switch II and α3-helix, which led to the loosening and opening of binding pocket. Finally, the coordinated motion of the switch II and α3-helix induced by the Y96D mutation further disrupted the interaction between key residues in the binding pocket and caused the increased solvent exposure of AMG510. These results elucidated the resistance mechanism on atomic level, which will help to offer guidance for the design of the next-generation of KRAS targeted drugs.

## Data availability statement

The original contributions presented in the study are included in the article/**Supplementary Material**. Further inquiries can be directed to the corresponding authors.

## Author contributions

Conceptualization, SL, YL and QW; methodology, HZhu and JF; validation, HZhu, JF, ML, HZha, XY and LL; formal analysis, HZhu and JF; investigation, HZhu and JF; writing—original draft preparation, HZhu and JF; writing—review and editing, SL and YL; visualization, HZhu, JF, ML, HZha, XY and LL; supervision, SL, YL and QW; project administration, SL, YL and QW; funding acquisition, XY All authors have read and agreed to the published version of the manuscript.

## Funding

This study was partly supported by grants from the Zhiyuan Scholar Program (Grant No. ZIRC2021-11) and UM Distinguished Visiting Scholar to XY.

## Conflict of interest

The authors declare that the research was conducted in the absence of any commercial or financial relationships that could be construed as a potential conflict of interest.

## Publisher’s note

All claims expressed in this article are solely those of the authors and do not necessarily represent those of their affiliated organizations, or those of the publisher, the editors and the reviewers. Any product that may be evaluated in this article, or claim that may be made by its manufacturer, is not guaranteed or endorsed by the publisher.
