# Supplementary material for: Mechanistic insights into the clinical Y96D mutation with acquired resistance to AMG510 in the KRASG12C
Source: Front Oncol. 2022 Aug 10;12:915512. doi: 10.3389/fonc.2022.915512 (PMC9399772; doi:10.3389/fonc.2022.915512)
Supplement: Supplementary file 1 [file DataSheet_1.docx]

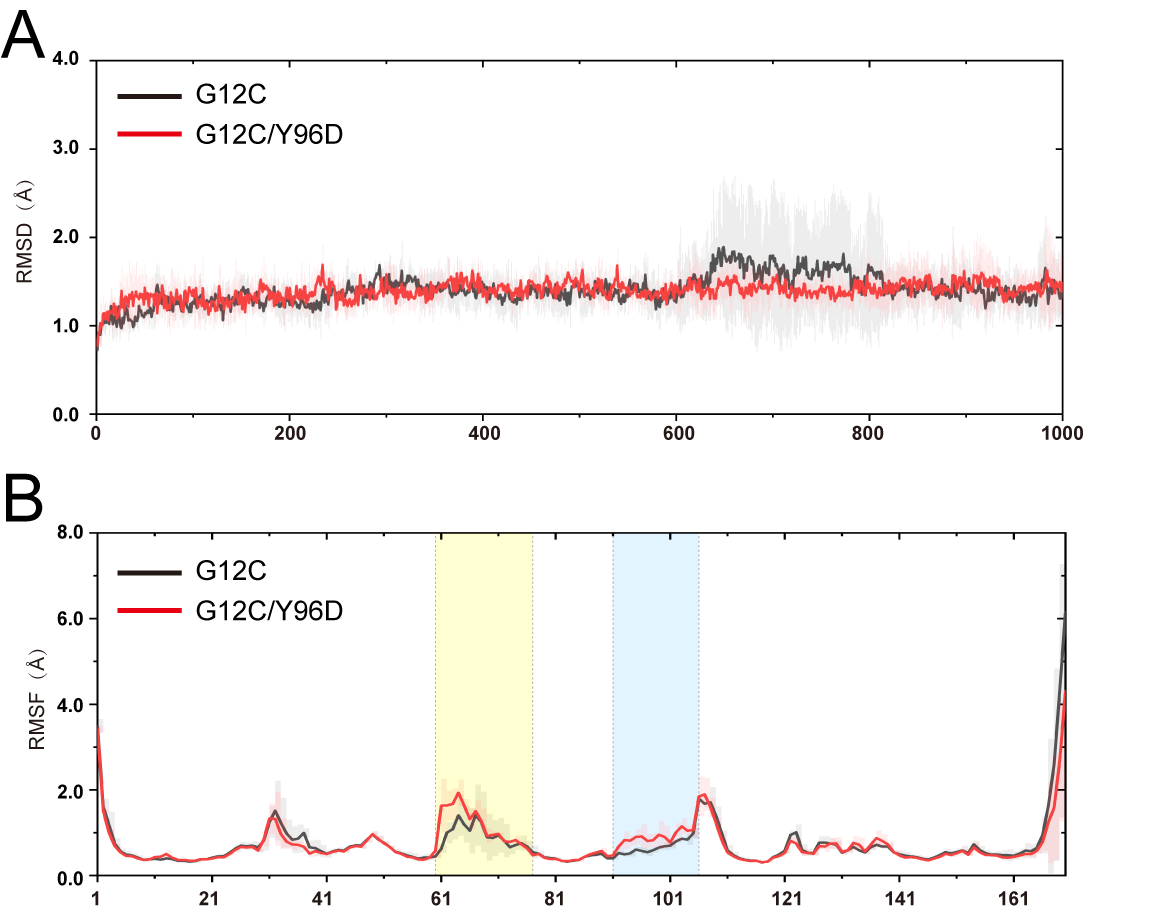


**Figure S1.** (A) Time evolution of the root-mean-square deviation (RMSD) of KRAS backbone atoms with respect to the initial structure in the G12C systems and the G12C/Y96D system. Black and red transparencies represent the SEM error for 3 replicas with respect to the average value. (B) Root-mean-square fluctuations (RMSF) of KRAS Cα atoms averaged over 3 independent runs in the G12C system and the G12C/Y96D system. Red and black transparencies represent the SEM error for 3 replicas with respect to the average value. The significant fluctuation differences are marked with yellow and blue backgrounds.

**
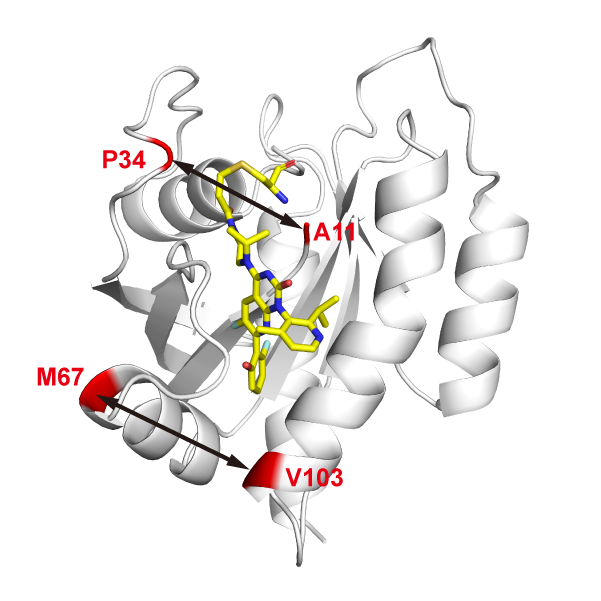
**

**Figure S2.** D_M67-V103_ represents the distance between switch II and α3-helix while d_A11-P34_ represents the distance between switch I and P-loop.


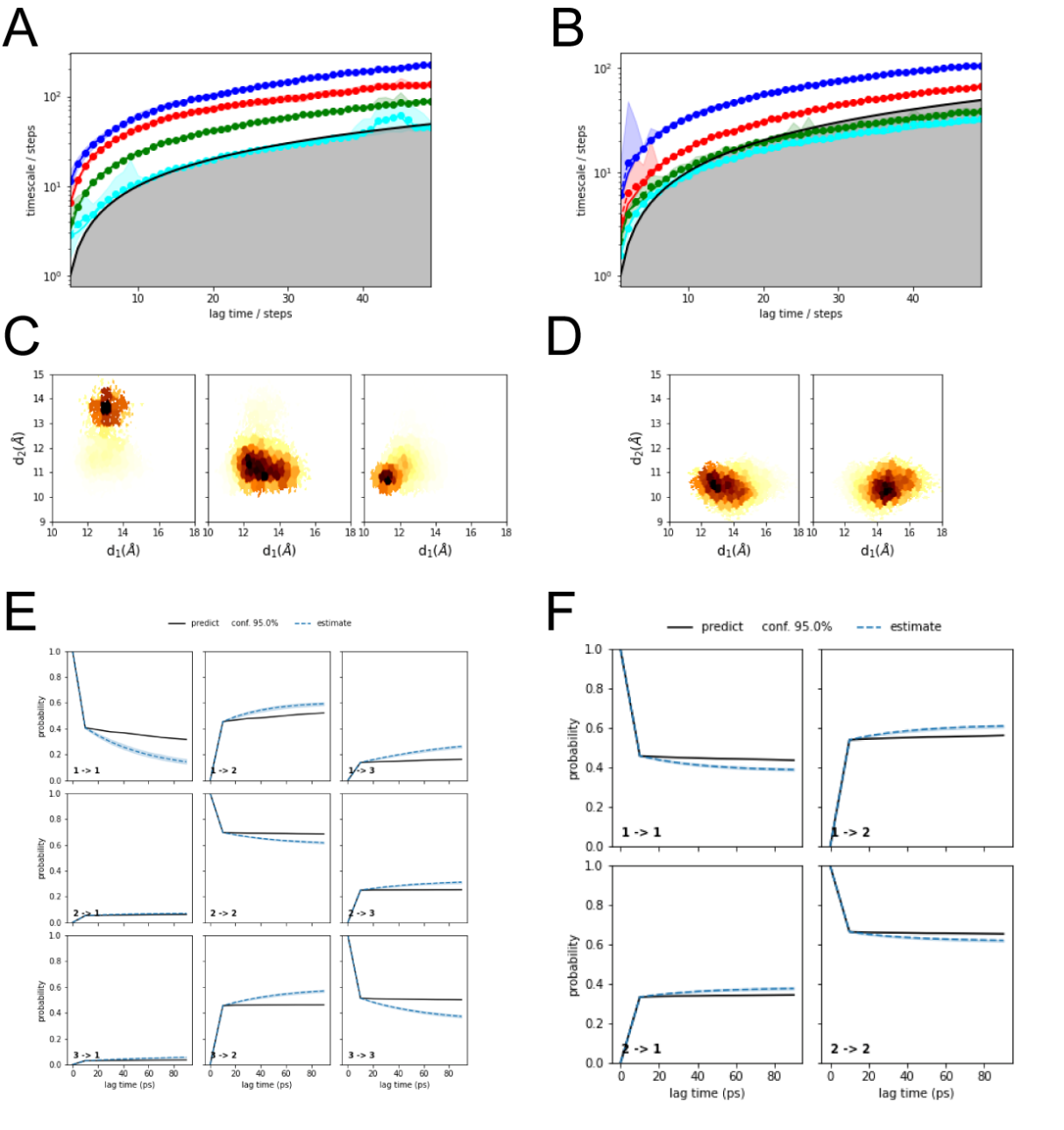


**Figure S3.** The MSM timescale test in the G12C system (A) and G12C/Y96D system (B). The clusters differentiated by MSM in the G12C system (C) and G12C/Y96D system (D). The MSM Chapman−Kolmogorov test in the G12C system (E) and the G12C/Y96D system (F).

*For only the states in the energy basin of free-energy landscape were analyzed, the first cluster in the G12C system was not analyzed and the two clusters differentiated by MSM in the G12C/Y96D system were combined.

**Figure S4.**
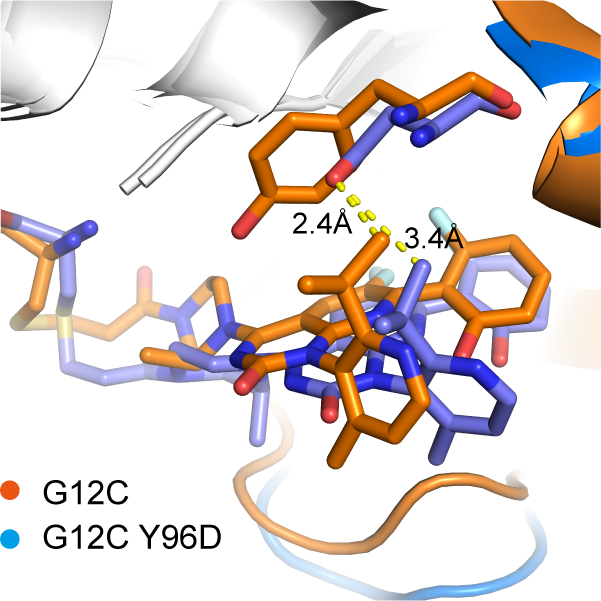
The distance between Asp96 (represented by the O atom of the carboxyl group) and the isopropyl group (represented by the Cβ atom) of AMG510 in the G12C system and the G12C/Y96D system.


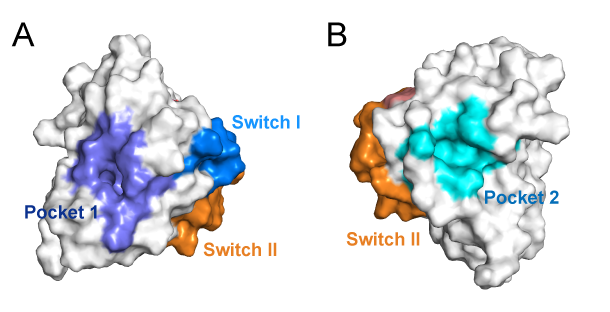
 **Figure S5.** Potential druggable pockets on KRAS surface. (A) Pocket 1 was composed of Y4, L6, L23, I24, N26, K42, Q43, V44, I46, L53, I55, R149, V152, D153, F156, Y157, L159, V160 and I163. (B) Pocket 2 was composed of V7, V9, F78, L79, C80, I93, R97, I100, K101, K104, D108, V109, M111, V112, L113, Y137 and I139.

**Table S1.** Occupancy of hydrogen bonds formed between switch II and α3-helix

| **Donor** | **Acceptor** | **G12C** | **G12C/Y96D** |
| --- | --- | --- | --- |
| Arg73@N | Val103@O | 31.67% | 5.40% |
| Arg102@N | Asp69@O | 28.20% | 0.00% |
| Lys104@N | Gly75@O | 47.43% | 48.15% |

**Table S2.** Occupancy of water-bridged hydrogen bonds in the two systems

| **Residue** | **G12C** | **G12C/Y96D** |
| --- | --- | --- |
| Gln99 | 28.43% | 23.60% |
| Gln61 | 11.70% | 7.40% |
| Tyr96/Asp96 | 0.97% | 20.35% |
| Lys16 | 0.00% | 19.10% |
| Thr58 | 10.00% | 10.55% |
